# Editorial: Integrating economics into population health: assessing policies and outcomes

**DOI:** 10.3389/fpubh.2026.1902201

**Published:** 2026-06-26

**Authors:** Dongyang Wang, Tie Yang, Ruipeng Song, Hang Yang, Tao Zhang, Chongtao Zhang

**Affiliations:** 1 Department of Nursing, The Third People's Hospital of Henan Province, Zhengzhou, China; 2 Information Center, The Third People's Hospital of Henan Province, Zhengzhou, China; 3 Vice Dean Office, The Third People's Hospital of Henan Province, Zhengzhou, China; 4 Faculty of Humanities and Social Sciences, Macao Polytechnic University, Macau, Macau SAR, China

**Keywords:** economics, editorial, outcomes, policies, population health

Under the contexts of deepening globalization, rapid demographic transitions, and more complex disease spectrums, the connotation and extension of public health are undergoing a profound shift. Modern population health has long broken through the traditional boundaries of biomedical and clinical interventions, evolving into a complex system deeply intertwined with policies, resource allocation, social determinants, and market mechanisms. Faced with increasingly tightening constraints on medical resources and continuously rising health demands, how to achieve the Pareto optimality of population health outcomes at the minimum social opportunity cost has become a severe challenge for global health policymakers.

The core objective and mission of this Research Topic, “*Integrating economics into population health: assessing policies and outcomes*,” is to construct an interdisciplinary analytical framework encompassing welfare economics, institutional economics, and econometrics to systematically and rigorously evaluate various health policies, interventions, and their health outcomes. To systematically address the multifaceted nature of population health, the 28 rigorously peer-reviewed studies collected in this Research Topic are conceptually woven into several interconnected domains (Table 1). Specifically, research on the Macroeconomic Determinants of Health explores the large-scale interplay between national economic growth, environmental externalities, and global health trajectories. At the micro and meso levels, Health Financing and Insurance investigates the risk-sharing mechanisms and payment reforms that shape healthcare markets, while Healthcare Resource Allocation and System Efficiency focuses on the spatial equity and operational performance of delivery systems under resource constraints. Furthermore, the Research Topic highlights Public Health Interventions and Economic Evaluation by applying cost-effectiveness analyses to assess specific policies and technologies. Finally, by delving into the Social Determinants of Health, the issue addresses the structural inequalities and systemic socio-economic burdens faced by vulnerable populations.

**Table 1:** Overview of the included articles.

Categories	Article type	Title
Macroeconomic Determinants of Health	Original Research	Heterogeneous associations of health expenditure, environmental pollution, and economic growth on life expectancy in BRICS economies
	Original Research	Distributional dynamics of child mortality in Africa: quantile evidence from economic, environmental, and demographic transitions.
	Original Research	Does the Health Kuznets Curve hypothesis hold for Saudi Arabia? A quantile ARDL analysis of disability-adjusted life years and their burden components.
	Original Research	Does the trade of medical products contribute to promoting human development?-An empirical analysis based on data from China and RCEP countries.
	Original Research	The impact of prudent financial policies on the urban-rural household health expenditure disparity: evidence from China.
Health Financing and Insurance	Original Research	Estimating the adverse selection and moral hazard in Urban and Rural Resident Basic Medical Insurance of China: a semi-parametric estimation approach
	Original Research	Indirect effects of long-term care insurance: does it affect the hospital expenditures of ineligible disabled individuals.
	Original Research	Determinants of willingness to pay for health insurance in later stages of the Covid-19 pandemic: findings based on the general adult population in Germany.
	Original Research	Analysis and optimization of inpatient cost structure for fracture patients under the implementation of the DRG policy.
	Original Research	China's coordinated tripartite medical reform: strategic balancing of interests among pharmaceuticals, healthcare, and health insurance.
	Original Research	Social medical insurance system and self-rated health: medical service utilization as the mechanism of action.
Healthcare Resource Allocation and System Efficiency	Systematic Review	Strategic resource allocation for malaria elimination in endemic settings: a systematic review of cost-effectiveness evidence.
	Original Research	Efficiency divergence and convergence in China's hospital sector: empirical insights from Guangdong Province using a multi-method approach.
	Original Research	Medicine affordability and access in India: lessons from generic-branded price variation under the Jan Aushadhi Scheme.
	Original Research	Structural imbalance of medical resources amid population mobility and digital empowerment: a study of national and port-developed provinces in China.
	Original Research	The effect of overuse by primary healthcare institutions on medical expenses: an empirical study from the western regions of China.
	Original Research	What are the key factors contributing to the inequity in healthcare resource allocation? Evidence from China's health panel data from 2009 to 2021.
Public Health Interventions and Economic Evaluation	Original Research	Market and welfare effects of a nationwide sugar-sweetened beverage tax in the U.S.
	Original Research	Does public spending on sports improve people's health? Empirical evidence from China.
	Original Research	From policy to practice: premarital spinal muscular atrophy screening as a public health initiative in northern Türkiye.
	Original Research	Estimation of the current healthcare costs of gout in Liaoning Province from 2015 to 2022 based on the SHA2011 accounting system.
	Original Research	Effect of “universal two-child” policy on population changes in Shandong province, China: an interrupted time series analysis.
	Original Research	Impact of social factors and health campaigns on the burden of idiopathic epilepsy: an inequality, decomposition, generalized and synthetic difference-in-differences study.
	Original Research	Four oral iron supplements for treating iron-deficiency anemia during pregnancy in China: a cost-effectiveness and budget analysis.
Social Determinants of Health	Scoping Review	Patient-centered burdens and economic outcomes among patients who are veterans, people with intellectual and developmental disabilities, and people living in rural areas and their caregivers: a scoping review
	Review	The economics of home support services in Ireland: exploring complex issues of healthcare sustainability and aging populations.
	Original Research	Social capital and health consciousness based on regional differences in China: a cross-sectional study.
	Original Research	Income inequality and multimorbidity patterns in China: a micro-level analysis using CHARLS.

## The heterogeneous game between macroeconomic externalities and health evolution

The non-linear dynamic relationship among macroeconomic growth, environmental evolution, and population health has always been the cornerstone of interdisciplinary research in health economics. When exploring the accumulation of health capital at the national level, one must confront the deep-seated game between the scale effects brought by economic expansion and the negative environmental externalities it induces (such as health depreciation caused by pollution).

Ridwan et al. provided rigorous cross-national empirical evidence for this Research Topic. Focusing on the BRICS economies, the study employs advanced panel data econometric models to deeply analyze the heterogeneous associations of health expenditure, environmental pollution, and economic growth on life expectancy. Traditional research often assumes that the promoting effect of economic growth on health is homogeneous; however, this study reveals through heterogeneous panel estimations that the marginal benefits of economic growth on life expectancy vary significantly under different stages of economic development and intensities of environmental regulation. This finding powerfully challenges the simplistic linear presumption that “growth equals health,” pointing out that during the critical transition period from developing to high-income countries, if strict controls on environmental externalities are not imposed, the health dividends brought by economic growth will be offset by environmental degradation. This study provides solid theoretical support for formulating macroeconomic policy combinations that balance economic vitality with population health.

## Information asymmetry, payment mechanism reshaping, and the healthcare services market

The inherent information asymmetry and principal-agent problems in the healthcare services market inevitably lead to market failure, which requires medical insurance systems and payment mechanisms to find a delicate balance between risk sharing and behavioral incentives.

In the micro-level game of the medical insurance market, adverse selection and moral hazard are two major stubborn diseases that weaken system efficiency. Wu and Xu applied an innovative semi-parametric estimation approach to successfully isolate and quantify these two effects within the complex empirical data of China's Urban and Rural Resident Basic Medical Insurance system. Compared with traditional fully parametric models, the semi-parametric method effectively avoids estimation biases caused by model misspecification, more accurately revealing the abnormal jump in healthcare service utilization after enrollees obtain insurance coverage. This research not only holds high demonstrative value in econometric methodology but also provides precise data navigation for policymakers to optimize premium actuarial pricing, dynamically adjust reimbursement rates, and design copayment mechanisms.

On the supply side, the reform of payment methods serves as the endogenous driving force for reshaping the behavior of healthcare institutions. Su et al. examined the evolution of inpatient cost structures for fracture patients under the implementation of the Diagnosis-Related Group (DRG) policy. As a prospective payment system, the core economic logic of DRGs lies in assigning the residual claim rights of cost control to hospitals, thereby incentivizing them to improve efficiency. However, the study astutely captured the alienation risks during policy implementation—through detailed data comparisons, the authors revealed that while DRGs effectively compress total expenses, they may trigger internal cost shifting and service structure distortions within hospitals. This finding emphasizes that when promoting DRG reforms, a refined regulatory system based on medical quality and clinical pathways must be established simultaneously.

## Public health interventions and cost-effectiveness evaluation from a welfare economics perspective

Based on the welfare economics framework, conducting ex-ante predictions and ex-post evaluations of specific policy interventions is a necessary closed loop for correcting market failures. Whether it is macro-level tax leverage or micro-level clinical medication guidance, rigorous economic justification is required.

Lee and Giannakas employed Pigouvian Tax theory to the public health domain, strictly quantifying the market equilibrium changes triggered by a nationwide sugar-sweetened beverage (SSB) tax in the U.S. The study constructed a structural equilibrium model encompassing heterogeneous consumer preferences and multi-oligopoly market competition, precisely measuring the tax pass-through rate and the offsetting effects between net welfare losses and long-term health gains across different income strata. Such in-depth welfare economics analysis surpasses simple public health advocacy, providing highly persuasive evidence for using price mechanisms to intervene in adverse health behaviors.

At the level of specific medical technology interventions, precise Cost-Effectiveness Analysis (CEA) is the core of Health Technology Assessment (HTA). Zhang et al. conducted a cost-effectiveness and budget impact analysis with high clinical translational value for four oral iron supplements treating iron-deficiency anemia during pregnancy in China. The study not only simulated the long-term health outcomes of different intervention strategies using Markov models but also incorporated real-world health system cost data, directly answering the practical question of “how to maximize utility between efficacy and cost.” This provides irrefutable evidence-based foundations for the dynamic adjustment of essential medicine lists.

## Structural imbalance of resource allocation, digital empowerment, and social determinants of health

The realization of health equity relies not only on the geographical accessibility of medical services but is also deeply rooted in the macro-resource distribution patterns and micro-social class structures.

Faced with rapid urbanization and population mobility, Fu and Lu utilized spatial econometric tools to systematically diagnose the structural imbalance of medical resource allocation in China. The study forward-lookingly introduced the variable of “digital empowerment,” profoundly analyzing the potential and limitations of digital technology in breaking geographical barriers and optimizing resource allocation. The research pointed out that although digital technologies like telemedicine have alleviated supply-demand contradictions in some areas, the siphon effect of populations still leads to significant polarization in medical resource density between core cities and peripheral regions. This conclusion points the direction for resource restructuring in the construction of regional medical centers and the deepening of tiered diagnosis and treatment systems.

In exploring micro-level Social Determinants of Health (SDOH), Ouyang et al. utilized high-quality microdata from the China Health and Retirement Longitudinal Study (CHARLS) to reveal the strong endogenous correlation between income inequality and multimorbidity patterns among middle-aged and older adults populations. Breaking through the limitations of single-disease research, the study applied advanced statistical methods such as Latent Class Analysis (LCA) to depict how socioeconomic status, through the cumulative effects of the life course, ultimately internalizes into complex pathological networks. This warns policymakers that the fundamental solution to the explosion of chronic diseases lies not merely in end-stage clinical salvage but in front-end income distribution adjustments.

Finally, addressing how macro policies can bridge this systemic health inequality, Zou et al. provided a policy evaluation case. By constructing a Difference-in-Differences (DID) model, the study robustly demonstrated the “reservoir” and “stabilizer” roles of prudent financial policies in narrowing the urban-rural household health expenditure disparity in China. It proved that through transfer payments and targeted financial investments, the polarization effects generated by market mechanisms in health resource allocation can be effectively hedged, thereby driving substantive progress in health equity at the national level.

## Conclusion

By bringing together 28 high-quality studies, this Research Topic fully demonstrates the powerful explanatory capability of modern economic methods in deconstructing the complexities of public health. These studies collectively corroborate a core paradigm: a highly resilient and sustainable public health system relies not only on the iteration of biomedical technologies but also on a resource allocation framework and incentive-compatible mechanisms built on scientific rationality. Looking ahead, we call on the academic community to further embrace data science, explore predictive health economics models based on artificial intelligence, and deepen interdisciplinary intervention research on the health poverty traps of vulnerable groups. Only by deeply integrating the rigorous logic of economics with the inclusive spirit of public health can we chart a fair, efficient, and sustainable evolutionary roadmap for global population health.

